# Supplementary material for: Research on the mechanical properties of EPS lightweight soil mixed with slag
Source: PLoS One. 2024 Jan 24;19(1):e0297372. doi: 10.1371/journal.pone.0297372 (PMC10842304; doi:10.1371/journal.pone.0297372)
Supplement: Attachment — Submitted filename: PONE-D-23-31063.pdf [file pone.0297372.s003.pdf]

PLOS ONE

Research on Mechanical Properties of Slag EPS Mixed Lightweight Soil

--Manuscript Draft--

**Manuscript Number:**	PONE-D-23-31063
**Article Type:**	Research Article
**Full Title:**	Research on Mechanical Properties of Slag EPS Mixed Lightweight Soil
**Short Title:**	1 Materials and methods<br>1.1 Material Selection<br>1.2 Specimen preparation
**Corresponding Author:**	Dali Xiang<br>Hubei University of Technology<br>wuhan, CHINA
**Keywords:**	Keywords: lightweight soil; EPS particles; slag; mechanical properties; deformation modulus; microscopic
**Abstract:**	In order to promote the wide application of lightweight soil in geotechnical engineering, the use of slag to replace part of the cement as a curing agent can better improve the effect of soil curing and reduce the cost of the project. The mechanical properties of Slag EPS Mixed Lightweight Soil were analyzed by conducting an unconfined compressive strength (UCS) test and triaxial test on lightweight soil with different EPS and slag content. The results show that the UCS decreased significantly with the increase of EPS content at different EPS particles and slag contents; the UCS of the specimen with 30% slag content is the largest, and the strength of lightweight soil without slag is comparable to that of lightweight soil with slag content of 60% or so; the peak stress in triaxial increases with the increase of the confining pressure, and the modulus of deformation decreases linearly with the increase of the content of EPS. The results show that: slag-cement composite curing agent has a significantly better reinforcing effect than single mixed cement; slag EPS mixed lightweight soil has two characteristics of hardening and softening, EPS particles, and slag content to determine the stress-strain characteristics of the lightweight soil; in the macro-mechanical properties of the study based on the microscopic mechanism of the slag EPS mixed lightweight soil analysis, different slag content affects the damage pattern of EPS mixed lightweight soils. The research results can provide a reference for engineering design and application.
**Order of Authors:**	Lifang Mei<br>Dali Xiang<br>Yiwen Huang
**Additional Information:**	
**Question**	**Response**
**Financial Disclosure**<br><br>Enter a financial disclosure statement that describes the sources of funding for the work included in this submission. Review the submission guidelines for detailed requirements. View published research articles from *PLOS ONE* for specific examples.<br><br>This statement is required for submission and **will appear in the published article** if	The author(s) received no specific funding for this work.

Powered by Editorial Manager® and ProduXion Manager® from Aries Systems Corporation

the submission is accepted. Please make sure it is accurate.<br><br>**Funded studies**<br>Enter a statement with the following details:<br>• Initials of the authors who received each award<br>• Grant numbers awarded to each author<br>• The full name of each funder<br>• URL of each funder website<br>• Did the sponsors or funders play any role in the study design, data collection and analysis, decision to publish, or preparation of the manuscript?<br><br>Did you receive funding for this work?	
**Competing Interests**<br><br>Use the instructions below to enter a competing interest statement for this submission. On behalf of all authors, disclose any competing interests that could be perceived to bias this work—acknowledging all financial support and any other relevant financial or non-financial competing interests.<br><br>This statement is required for submission and **will appear in the published article** if the submission is accepted. Please make sure it is accurate and that any funding sources listed in your Funding Information later in the submission form are also declared in your Financial Disclosure statement.<br><br>View published research articles from *PLOS ONE* for specific examples.	The authors have declared that no competing interests exist.

Powered by Editorial Manager® and ProduXion Manager® from Aries Systems Corporation

**NO authors have competing interests**<br><br>Enter: *The authors have declared that no competing interests exist.*<br><br>**Authors with competing interests**<br><br>Enter competing interest details beginning with this statement:<br><br>*I have read the journal's policy and the authors of this manuscript have the following competing interests: [insert competing interests here]*<br><br>* typeset	
**Ethics Statement**<br><br>Enter an ethics statement for this submission. This statement is required if the study involved:<br><br>• Human participants<br>• Human specimens or tissue<br>• Vertebrate animals or cephalopods<br>• Vertebrate embryos or tissues<br>• Field research<br><br>Write "N/A" if the submission does not require an ethics statement.<br><br>General guidance is provided below. Consult the submission guidelines for detailed instructions. **Make sure that all information entered here is included in the Methods section of the manuscript.**	N/A

Powered by Editorial Manager® and ProduXion Manager® from Aries Systems Corporation

**Format for specific study types**<br><br>**Human Subject Research (involving human participants and/or tissue)**<br>• Give the name of the institutional review board or ethics committee that approved the study<br>• Include the approval number and/or a statement indicating approval of this research<br>• Indicate the form of consent obtained (written/oral) or the reason that consent was not obtained (e.g. the data were analyzed anonymously)<br><br>**Animal Research (involving vertebrate animals, embryos or tissues)**<br>• Provide the name of the Institutional Animal Care and Use Committee (IACUC) or other relevant ethics board that reviewed the study protocol, and indicate whether they approved this research or granted a formal waiver of ethical approval<br>• Include an approval number if one was obtained<br>• If the study involved *non-human primates*, add *additional details* about animal welfare and steps taken to ameliorate suffering<br>• If anesthesia, euthanasia, or any kind of animal sacrifice is part of the study, include briefly which substances and/or methods were applied<br><br>**Field Research**<br><br>Include the following details if this study involves the collection of plant, animal, or other materials from a natural setting:<br>• Field permit number<br>• Name of the institution or relevant body that granted permission	
**Data Availability**<br><br>Authors are required to make all data underlying the findings described fully available, without restriction, and from the time of publication. PLOS allows rare exceptions to address legal and ethical concerns. See the PLOS Data Policy and FAQ for detailed information.	Yes - all data are fully available without restriction

A Data Availability Statement describing where the data can be found is required at submission. Your answers to this question constitute the Data Availability Statement and **will be published in the article**, if accepted.<br><br>**Important:** Stating 'data available on request from the author' is not sufficient. If your data are only available upon request, select 'No' for the first question and explain your exceptional situation in the text box.<br><br>Do the authors confirm that all data underlying the findings described in their manuscript are fully available without restriction?	
**Describe where the data may be found in full sentences. If you are copying our sample text, replace any instances of XXX with the appropriate details.**<br><br>• If the data are **held or will be held in a public repository**, include URLs, accession numbers or DOIs. If this information will only be available after acceptance, indicate this by ticking the box below. For example: *All XXX files are available from the XXX database (accession number(s) XXX, XXX.*).<br>• If the data are all contained **within the manuscript and/or Supporting Information files**, enter the following: *All relevant data are within the manuscript and its Supporting Information files.*<br>• If neither of these applies but you are able to provide **details of access elsewhere**, with or without limitations, please do so. For example:<br><br>*Data cannot be shared publicly because of [XXX]. Data are available from the XXX Institutional Data Access / Ethics Committee (contact via XXX) for researchers who meet the criteria for access to confidential data.*<br><br>*The data underlying the results presented in the study are available from (include the name of the third party*	All relevant data are within the manuscript and its Supporting Information files.

Powered by Editorial Manager® and ProduXion Manager® from Aries Systems Corporation

and contact information or URL).<br>• This text is appropriate if the data are owned by a third party and authors do not have permission to share the data.<br><br>* typeset	
Additional data availability information:	

Research on Mechanical Properties of Slag EPS Mixed Lightweight Soil

Click here to access/download;Manuscript;PLoS one.docx

Research on Mechanical Properties of Slag EPS Mixed Lightweight Soil

Lifang Mei[1,2], Dali Xiang [1*], Yiwen Huang [1]

1 School of Civil Engineer and Architecture, Hubei University of Technology, Wuhan 430068，China, **2** Hubei Research Center of Ecological Road Engineering, Wuhan 430068，China

* 102110966@hbut.edu.cn

Abstract

I[illegible]der to promote the wide application of lightweight soil in geotechnical engineering, the use of slag to replace part of the cement as a curing agent can better improve the effect of soil curing and reduce the cost of the project. The mechanical properties of Slag EPS Mi[illegible]ightweight Soil were analyzed by conducting an unconfined compressive strength (UCS) test and triaxial test on lightweight soil with different EPS and slag content. The results show that the UCS decreased significantly with the increase of EPS content at different EP[illegible]rticles and slag contents; the UCS of the specimen with 30% slag content is the largest, and the strength of lightweight soil without slag is comparable to that of lightweight soil with slag content of 60% or so; the peak stress in triaxial increases with the increase of the confining pressure, and the modulus of deformation decreases linearly with the increase of the content of EPS. The results show that: slag-cement composite curing agent has a significantly better reinforcing effect than single mixed cement; slag EPS mixed lightweight soil has two characteristics of hardening and softening, EPS particles, and slag content to determine the stress-strain characteristics of the lightweight soil; in the macro-mechanical properties of the study based on the microscopic mechanism of the slag EPS mixed lightweight soil analysis, different slag content affects the damage pattern of EPS mixed lightweight soils. The research results can provide a reference for engineering design and application.

Introduction

With the rapid advancement of modern industry, the utilization of polymer materials is increasingly widespread, polystyrene foam (EPS) is a new type of geotechnical material produced in this context. the research and application of EPS mixed lightweight soil in foreign [illegible]ntries began in the 1970s, Some scholars have carried out a large number of theoretical studies on it, and have now achieved certain resu[illegible]Currently, some countries [illegible] more applications in this area, and there are already some examples of research and application to engineering projects [1-4], such as weak foundations, highway settlement, embankment filling, stabilizing slopes, bridge abutments

and backfilling of underground vo[illegible]. The density of EPS hybrid lightweight soils is small compared to that of normal soils [5,6], and the EPS particle content is the main factor determining the density magnitude of lightweight soils, with the cement content having a lesser effect. Lightweight soil as a geosynthetic material, the content of EPS particles and curing agent is the main factor affecting the compressive strength of this material, other factors have less influence on the strength, and the mechanical properties mainly depend on the proportion of the lightweight soil mixture [7-9].

Studies on the mass incorporation of slag into EPS lightweight soils are rare, and the most commonly used curing agent [illegible]ement, but the production of cement ha[illegible]ious environmental problems and consumes a large amount of energy in the production process [10]. Slag, as an industrial by-product from a wide range of sources, is relatively inexpensive, and the use of slag instead of cement as a curing agent can both reduce the cost of foundation treatment and minimize the impact on the environment [11,12]. Slag, as an industrial waste material, has been widely used in concrete projects, and by replacing part of the cement in the concrete mix with slag, the construction cost of raw material[illegible] related energy costs have been reduced, and the mechanical properties and durability of concrete have been significantly improved [13-15]. Zhao et al. [16] and others used cement and slag to cure coastal saline soils, and the curin[illegible]ect was better than that of cement alone; Liang [17] , and Nu et al. [18] conducted indoor tests to show that: replacing part of the cement with slag can substantially increase the compressive strength of hydraulic soils, and it was found that increasing the amount of slag admixture can improve strength of hydraulic soils. Slag is used in the field of geotechnical engineering, utilizing the lightweight properties of EPS to form slag EPS hybrid lightweight soil, which not only eliminates waste slag but also produces a lightweight, high-strength fill material [1[illegible]].

In this study, the clay in Wuhan, Hubei Province is used as the raw material soil, EPS particles as the lightweight material, slag, and cement are mixed, different slag mixing amount configu[illegible]on mixing curing agent, a variety of materials mixed to form slag EPS mixed lightweight soil, and the mechanical properties of slag EPS mixed lightweight soil are investigated under different ratios. Due to the addition of lightweight materials and curing agents, its unique material structure composition, resulting in slag EPS mixed with lightweight soil physical and mechanical properties are very complex, so it is necessary to research the mechanical properties of lightweight soil. Mechanical properties and destruction morphology of slag EPS mixed lightweight soils were investigated by unconfined compressive strength (UCS) test and consolidated undrained triaxial (CU) test, the use of scanning electron microscopy to study its microstructure, to explore the macro-mechanical mechanism, and its research results can provide a reference for engineering design and application.

1 Materials and methods

1.1 Material Selection

The raw material soil used in the test is clay, taken from a project pit in Wuhan City, The depth of the soil is about 5m below the ground, and its basic physical parameters are shown in Table 1. The lightweight materials used EPS spherical particles, diameter 1 ~ 3mm, pure particle density of 0.024g/cm3, and packing density of 0.016g/cm3. curing agent used 42.5 ordinary silicate cement and s95 granulated slag, the chemical composition of the raw materials is shown in Table 2. Before

the test, the retrieved soil specimens were dried at a temperature range of 105~110°C for a period of not less than 8 h. The dry soil was crushed and passed through a sieve of 1 mm aperture, and then the sieved dry soil was put into a plastic bag and sealed.

Table 1. Physical-mechanical parameters of raw soil

Density ρ/g·cm^{-3}	Void ratio e	Water content *w*/%	Plastic limit W_P/%	Plasticity index I_p	Liquid limit W_L/%	Specific gravity Gs
1.917	0.544	21.79	20.29	19	38.79	2.64

Table 2. Chemical compositions of cement and Slag *w*/%

Composition	CaO	Fe_2O_3	SiO_2	Al_2O_3	MgO	SO_3	Loss
Cement	61.23	3.28	22.56	6.25	2.28	1.67	1.02
Slag	34	1.03	34.5	17.7	6.01	1.64	0.84

1.2 Specimen preparation

The ratio of specimens will be based on a mass ratio standard (ratio of admixture of other materials to the mass of dry soil). The EPS particles were mixed at 1%, 2%, 3%, and 4%, and the slag-cement composite curing agent was mixed at 10%, 15%, 20% and 25% respectively. The numerical value of slag as a percentage of curing agent was 0%, 10%, 30%, 50%, and 70% and water was tap water.

According to a certain ratio of weighing the appropriate amount of dry soil, slag, and cement, placed in the sampling barrel fully mixed, according to 50% water content, mixed with water, stirred into a homogeneous mixed slurry, and then add EPS particles, stirring for 10 minutes to form a homogeneous slag EPS mixture of lightweight soil (for the convenience of the narrative can be referred to as lightweight soil). The lightweight soil was in 3 layers into a three-flap mold (diameter 39. l mm, height 80 mm), each layer hit 25, Turning with a soil scraper between each layer and to a depth of not less than 1cm, until the formation of a dense specimen. Finally, the prepared specimen together with the specimen maker into the standard curing box, curing temperature (20 ± 2) ℃, relative humidity greater than 95%, after 24h of curing demolding, and continued to curing to the design age of 28 d. Each group of tests to produce three parallel specimens, take the average as the final results.

In this paper, the above lightweight soil specimens were tested for UCS concerning the Standard for Geotechnical Test Methods [21], and the instrument used was the WDW-10E microcomputer-controlled electronic universal testing machine, with a strain rate of 1 mm/min. The CU triaxial compression test was carried out with a strain-controlled triaxial compression apparatus, which was taken to have a strain rate of 0.08 mm/min. The confining pressures of the test were taken as 50, 100, 200, and 300 kPa respectively. During microscopic testing, the test specimens were cut into thin slices for drying after reaching the curing period. After that, the specimens were broken into small squares, and 3 specimens were taken from each specimen, and the specimens were coated. The scanning electron microscope technique was used to analyze the microstructure of the lightweight soil.

2 Results and Discussion

2.1 Effect of EPS content on the density of lightweight soil

EPS particles are a kind of lightweight material with good performance, which can be added into the soil body to significantly reduce the load of the soil body itself. [illegible]e test to consider the effect of EPS particles and curing agent on the density, EPS particles content was 1%, 2%, 3%, and 4%, and curing agent content was 10%, 15%, 20%, and 25%, the ratio of slag to cement was taken as 1:1, a total of 16 groups of specimens, and the average of the density of the lightweight soil after 28d of maintenance was analyzed in the test. Figure 1 shows the relationship between different EPS particles, curing agent mixing amount, and lightweight soil. From Figure 1, it can be seen that: With the changes in curing agent and EPS content, the density of lightweight soil is the minimum of 0.841g/cm^3 (curing agent content is 10%, EPS content is 4%), and the density is the maximum of 1.49g/cm^3 (curing agent content is 25%, EPS content is 1%); When the EPS particle content is unchanged, with the increase of curing agent content, the trend of density change is not obvious, slightly increased, the density difference between the adjacent curing agent content changes only 0.096 ~ 0.112g/cm^3, which can be seen that the curing agent content on the density of the effect is very small; When the amount of curing agent is unchanged, the density decreases significantly with the increase of EPS content, and the density decreases by at least 20% for every 1% increase in the content of EPS particles. But from the EPS content increases from 1% to 2%, the density decrease is the largest. Taking the curing agent addition of 10% as an example, when the EPS content increases from 1% to 4%, the density decreases from 1.436 g/cm^3 to 0.841 g/cm^3, which is a reduction of 41.5%, indicating that the density of the lightweight soil decreases significantly. This law shows that it is feasible to realize the lightweight of lightweight soil by adding EPS particles, thus it can be considered that the density of lightweight soil is mainly affected by the content of EPS particles, and the content of curing agent is not the main influence factor of density.

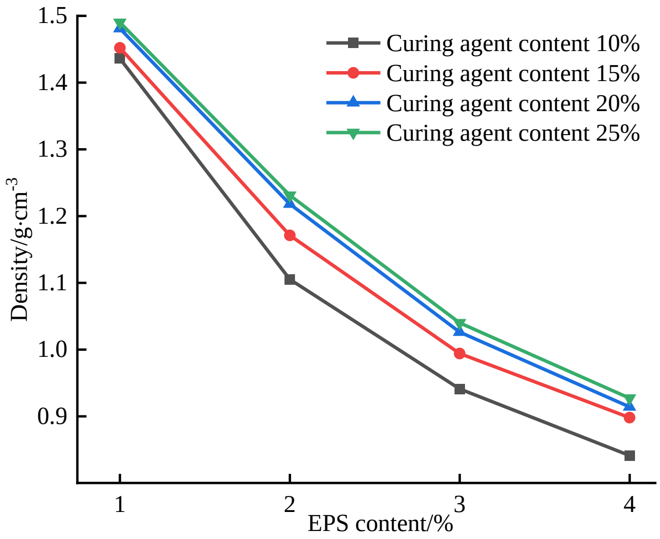

Fig 1. Density versus EPS content

EPS particles can not be unlimited increased in the amount of mixing, The density of lightweight soil cannot be unlimited reduced, and the excess of EPS particles leads to a reduction of the bonding

effect of the curing agent in them, it is difficult to make a specimen, the lightweight soil to lose the value of the practical application.

2.2 Effect of EPS content on unconfined compressive strength

The addition of lightweight materials and curing agents results in lightweight soils that are different from natural soils, but instead resemble porous cemented soils. The truly novel aspect of this geomaterial is the inclusion of lightweight materials that create a large number of cavity structures within the soil mix, thereby greatly reducing its weight, but still providing a certain level of strength. In Fig. 2, the content of the curing agent is 10%, 15%, 20%, and 25%, the content of EPS particles is 1%, 2%, 3%, and 4%, and the ratio of slag to cement is 1:1, and the UCS curves of a total of 16 groups of specimens are taken. The UCS of different EPS content has a similar trend, with the increase of EPS content, the strength is decreasing. Especially when the EPS content is less than 2%, the strength decreases more, for example, when the content of the curing agent is 25% when the EPS content is increased from 1% to 2%, the strength decreases from 1464.1 kPa to 600.5 kPa, which is 58.9%; when the EPS content is more than 3%, the strength also decreases significantly, but the trend of decrease is slower; when the EPS content is 4%, the strength is 205.7kPa, which is reduced by 85.9%. Therefore, weight can not be reduced by increasing the amount of EPS without limitation, which will have a greater impact on the compressive strength.

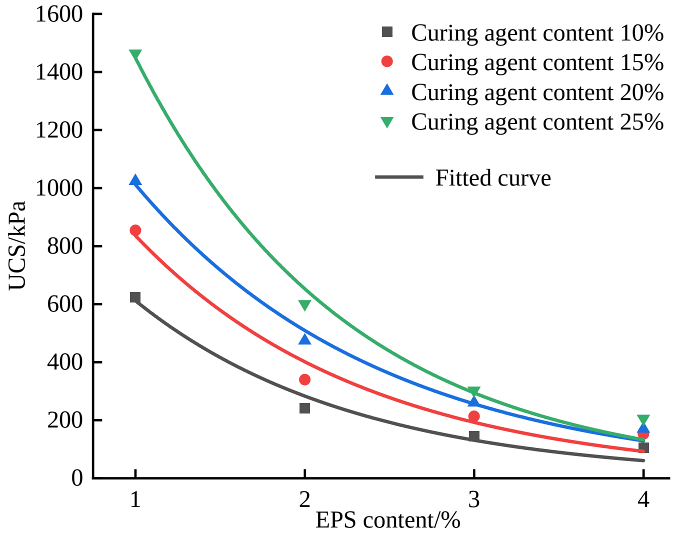

Fig 2. Relationship between UCS and EPS content

Fitting the data points in Fig. 2 reveals that the exponential curve fits well with a correlation coefficient R^2 greater than 0.96, which agrees with the findings of Mei [22] on lightweight soils, where the relationship between the UCS and the EPS admixture is：

$$q_{\mathrm{u}} = q_0 \mathrm{e}^{-t a_{\mathrm{e}}} \quad (1)$$

In formula (1): q_{u} is the UCS； a_e is the EPS content; q_0 and t are the fitting parameters for the relationship between strength and EPS content, respectively, and are related to the curing agent content. It can be seen that both q_0 and t increase with the increase of curing agent content.

2.3 Effect of curing agent content on unconfined compressive

strength

The EPS content was selected as 1%, and the relationship curves between different curing agent content and UCS were plotted, and the slag content of the specimens in Fig. 3 was 0%, 10%, 30%, 50%, and 70%, and the curing agent content was 10%, 15%, 20%, and 25%, respectively. When the slag content was constant, the UCS at different curing agent contents had the same trend, the strength gradually increased with the increase of curing agent content. The UCS at 25% curing agent content was 1.96, 2.14, 2.15, 2.35, 2.56 times the UCS of the same group of specimens with 10% curing agent content. Slag content only affects the slope and intercept of the linear relationship. Fitting the data points in Figure 3, the correlation coefficient R^2 is greater than 0.93, which is a good fit. This is consistent with the findings of HOU [23] on lightweight soils. The relationship between the UCS and the content of the curing agent is:

$$q_u = ka_c + b \tag{2}$$

In formula (2): a_c is the curing agent content; k and b are both fitting parameters for the relationship between strength and curing agent content, which is related to slag content.

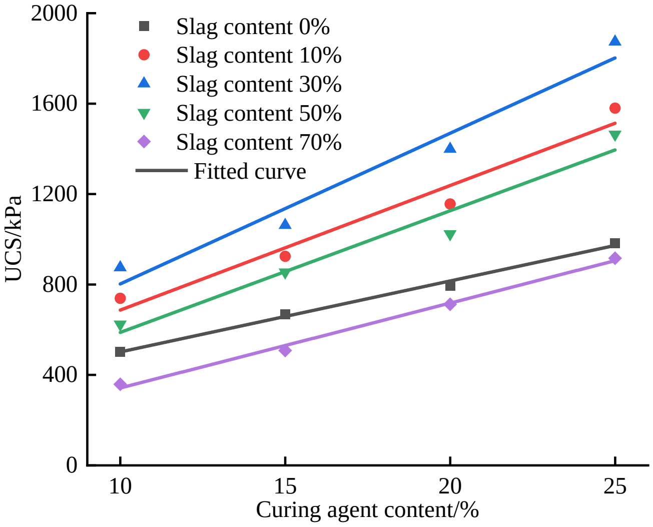

Fig 3. Relationship between UCS and curing agent content

2.4 Effect of slag content on unconfined compressive strength

The curing agent content was selected to be 20% unchanged, and the slag content was 0%, 10%, 30%, 50%, and 70%, and it can be seen from Fig. 4 that with the increase of slag content, the UCS curves of the lightweight soils increased and then decreased, which indicates that the content of slag is not the more the better. At the same EPS content, the UCS of the specimens reached the maximum value at 30% of slag content; when the EPS content was 1%, 2%, 3%, and 4%, compared with the specimens without slag content (curing agent were cement), the UCS of the specimens at 30% of slag content increased by 534.9 kPa, 400.7 kPa, 360.4 kPa and 212.4 kPa, respectively. The strength of the specimens without slag content is comparable to that of the specimens with about 60% slag content. This shows that: It is feasible to utilize slag to partially replace cement as a curing agent, but the amount of replacement should be within a suitable range. Compared with the lightweight soil without slag, the compressive strength of the lightweight soil with a certain percentage of slag is greater than that without slag at the same EPS content.

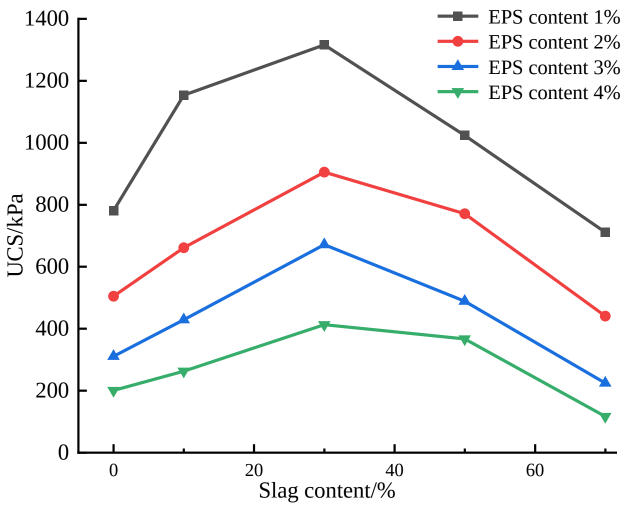

Fig 4. Relationship between UCS and slag content

2.5 Triaxial test analysis

The cured specimens were vacuum saturated for 2h, and after 24h of immersion in water, it was not fully saturated, and the saturation degree was 60% to 85%, and the test continued to be saturated with counterpressure in the triaxial instrument. The specific test program is shown in Table 3.

Table 3. Triaxial compression test (CU) scheme of lightweight soil

sch[illegible]	EPS content %	Slag content %	Water content %	Curing agent content %	Confining pressure kPa
1	2、3、4	30	50	20	50、100、200、300
2	3	10、30、50	50	20	50、100、200、300

The stress-strain curve obtained from the test is shown in Fig. 5. Where σ_1 is the large principal stress, σ_3 is the small principal stress, ε_d is the axial strain, and a_g is the slag content. From Figure 5, it can be seen that: in the case of the same amount of each content, the peak principal stress difference（$\sigma_1 - \sigma_3$）increases with the increase of the confining pressure, the stress-strain curve under different confining pressure has nonlinear characteristics, with the increase of strain, the slope of the curve becomes smaller, and the overall performance of strain hardening characteristics; when the confining pressure is the same, the strength decreases with an increase in EPS particle content, and the curve morphology is transformed from the softening type to the hardening type; In the case of the same amount of EPS content, the strength is affected by slag content, and 30% of slag content has the highest strength, which indicates that the ratio has a very important influence on the stress-strain relationship; Fig. 5(e) in the test with 3% EPS content and 50% slag content, the curves are of the softening type when the confining pressure is 50kPa and 100kPa, indicating that the specimens with stronger cemented structure exhibit softening and hardening characteristics when the confining pressure is at the relatively low level. lower level exhibits softening characteristics, and it is not an accidental phenomenon, similar test phenomena can be observed from the research data of literature [24].

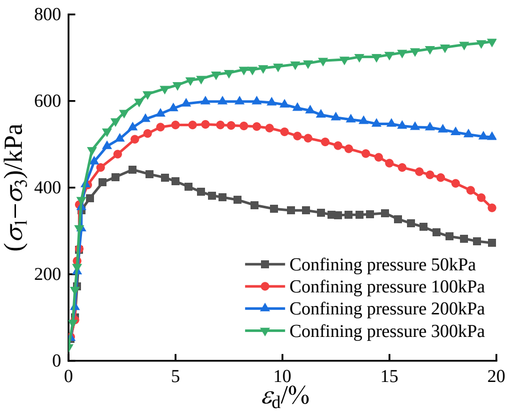

(a) a_e =2%, a_g =30%

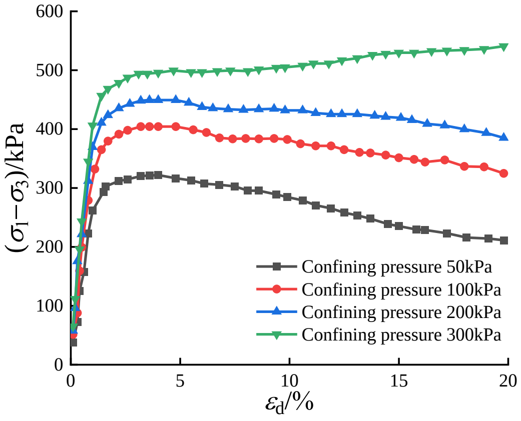

(b) a_e =3%, a_g =30%

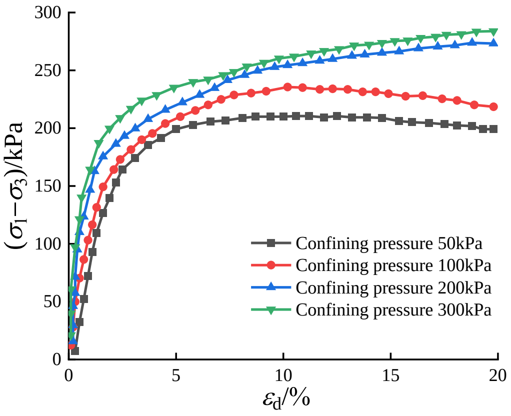

(c) a_e =4%, a_g =30%

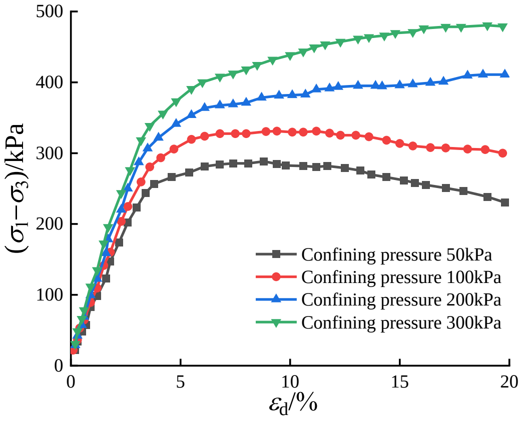

(d) a_e =3%, a_g =10%

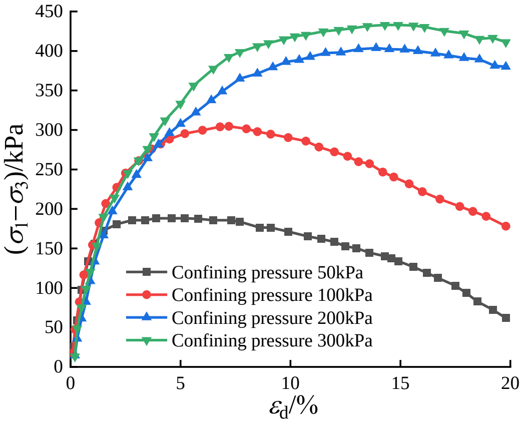

(e) a_e =3%, a_g =50%

Fig 5. Stress-strain relationship curves of lightweight soils with different ratios

From the test results, the initial stage of the stress-strain relationship curve of lightweight soil is a straight line and elastic deformation occurs. After reaching the yield stress, the specimen undergoes elastic-plastic deformation, and the stress-strain relationship shows nonlinear characteristics. The research results of Pei [25] and others showed that the stress-strain relationship

of the more structural clay was strain-hardening when the confining pressure was higher than the structural yield stress, and strain-softening when the confining pressure was lower than the structural yield stress. Hou [26] used silt to prepare lightweight soil, and conducted a CU test to obtain the result that when the confining pressure is less than the structural yield stress of the lightweight soil specimen, the strain softening phenomenon is more obvious; when the confining pressure is greater than the structural yield stress of the specimen, the strain hardening phenomenon is more obvious. Although a large number of EPS particles were mixed in, the particles of lightweight soil were combined very tightly due to the net-like cementation structure formed by the curing effect of the curing agent, and entered the recompression and compacting stage under the high confining pressure, showing a tendency of increasing strength. On the contrary, under the limiting effect of low confining pressure, the stress of lightweight soil increases with strain to a certain degree and then slowly decreases to a certain residual strength, and the stress-strain curve exhibits a hump curve, i.e., strain softening type. While Dong et al. [27] used Nanjing silty powdery clay as raw material soil to make lightweight soil specimens (under the condition of comparable ratios), the CU test curve patterns were all of the strain-hardening type, which indicates that the transformation of the stress-strain relationship of the lightweight soils and the type and nature of the raw material soils also have a certain relationship.

2.6 Analysis of factors affecting the deformation modulus of lightweight soils

Due to the compression and shear expansion of the soil, the modulus of deformation E is not a constant, but a parameter that varies with the stress level. The average deformation modulus E_{50}, which is the slope of the cut line of the stress-strain curve from 0 to 50% of the compressive strength, is usually used in engineering, and is calculated as follows:

$$E = \frac{1/2\sigma_{max}}{\varepsilon_{1/2}} \quad (3)$$

In formula (3): σ_{max} is the compressive strength and $\varepsilon_{1/2}$ is the strain corresponding to 50% of the compressive strength.

The average deformation modulus of the soil body is related to the EPS particle content, curing agent content, and confining pressure. To simplify the problem, other factors were fixed, and linear regression analysis was performed on a single factor, and the correlation coefficient $R^2 = 0.958$, and the deformation modulus of specimens with different EPS admixture and different enclosing pressures showed a good normalization. As shown in Fig. 6. The deformation modulus of lightweight soil decreased with the increase of EPS content, and the discrete points were fitted, and the results showed that there was a linear relationship between the two, with the relationship equation as follows.

$$\mathrm{E} = \mathrm{k}a_e + c \quad (4)$$

In formula (4): k =-0.302, c =2.225, a_e is the EPS particle content, the deformation modulus is important for the study of soil subsidence and foundation settlement.

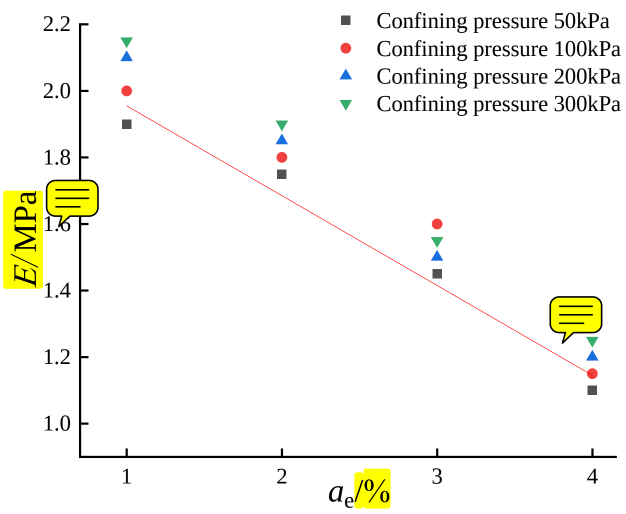

Fig 6. Relationship between deformation modulus and EPS content

2.7 Unconfined Compression Destruction Patterns

The destructio[illegible]he lightweight soil specimen reflects the structural nature of the soil body to a certain extent. For the analysis of the results of the UCS test, it was concluded that the specimens had two destruction modes cracking destruction and shear destruction, and the specimens had obvious rupture surfaces. The representative specimens were selected based on a curing agent content of 20% and an EPS content of 2%. Fig. 7(a) The specimen has an obvious rupture surface, the test has dropped blocks or loose broken phenomenon, and the specimen is cracking destruction. Figure 7 (b) specimen visible oblique surface is shear destruction, rupture surface inclination is slightly slower, the destruction of multiple cracks. Figure 7(c) specimen strength is higher, the pressure produces a penetrating crack, the shear band is obvious, and the rupture surface inclination (and the large principal stress surface angle) is very steep, showing shear destruction. Figure 7(d) and Figure 7(a) of the destruction pattern are the same, the specimen destruction appears bulging and belongs to the cracking destruction.

Through observation and analysis, under the action of external load, the holes inside the specimen and the interface of EPS particles are the first to experience stress concentration, and the shear band cuts through these parts, and the EPS particles themselves do not undergo shear destruction, but from the rupture surface macroscopically, it can be observed that the EPS particles undergo a certain degree of deformation. The specimens showed different destruction patterns depending on the content of slag and cement. The lightweight soil without slag was easy to be loose and broken, and after adding an appropriate amount of slag, the structural and mechanical properties of lightweight soil were enhanced.

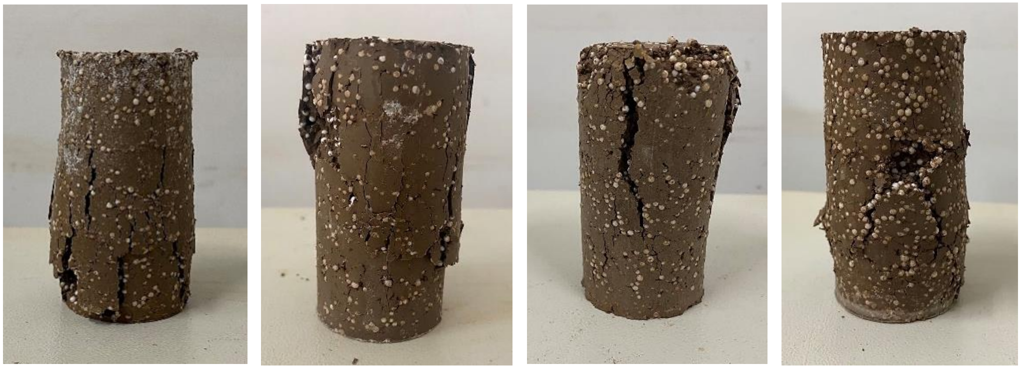

(a) (b) (c) (d)

a—0%, b—10%, c—30% , d—50%

Fig 7. Damage patterns of specimens with different slag contents

2.8 Characterization of the microstructure of lightweight soils

The physic mechanical properties of soil are essentially controlled by its internal structure, and any of its complex physic mechanical traits are the comprehensive manifestation of microstructural properties and their changes, and the mechanism of macroscopic mechanical behavior of soil can be understood through the in-depth study of the microstructural characteristics of the soil body [28,29]. To further study the slag-cement-cured lightweight soil, scanning electron microscope SEM observation of lightweight soil with different content was carried out to analyze the microstructure of lightweight soil with different compositions of curing agent action through electron micrographs. The specimens with 1% EPS particles and 20% curing agent content were selected for observation.

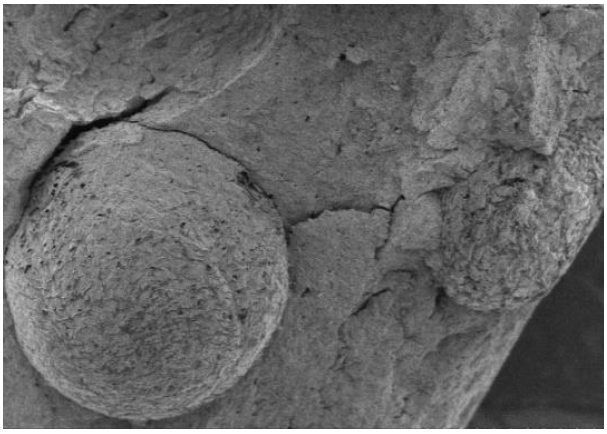

Fig 8. EPS microscopic morphology

Figure 8 is a microscopic picture of the combination of mixed soil and EPS particles, the surface of the EPS particles is wrapped with a large number of cements, which connects the EPS particles and soil particles tightly. The EPS particles have a hollow honeycomb structure, with a large number of pores, and therefore are light in mass, and the joint action with the curing agent makes the soil with lightweight and high-strength characteristics.

As seen in Figure 9a, the inter-particle pores of the slag-free specimen are more, and the cement in the soil body undergoes a hydration reaction, resulting in flaky and reticulated cement. Figure 9b with the slag into the specimen inter-particle pores filled by particles smaller slag particles, Lightweight soil contains a large number of crystals, crystals are more net-like, sheet distribution in the soil, and structural units are more agglomerated structure, the formation of a certain skeleton

filling effect, the soil structure of the skeleton of the degree of densification, can be seen curing agent plays a role in the soil as a colloid, so that the soil particles of the linkage between It can be seen that the hardener plays the role of cement in the soil body, which changes the type of association between soil particles from contact association to cementation association, and the cementation association strengthens the association strength between soil particles, so that the structural strength of the soil body shows an increasing trend. As shown in Figure 9c, with the increase of slag content, the skeleton filling effect is more obvious, the pore space between soil particles is smaller, the soil body is more compact, and the generation of cement is also gradually increased, the cement is wrapped around the soil particles to bond so that the macroscopic mechanical properties are greatly improved. It can be seen from Figure 9d: the more slag content, the generation of cement in the soil increases significantly, but the pore space is, the lightweight soil structure becomes loose instead, and the structural strength becomes weaker.

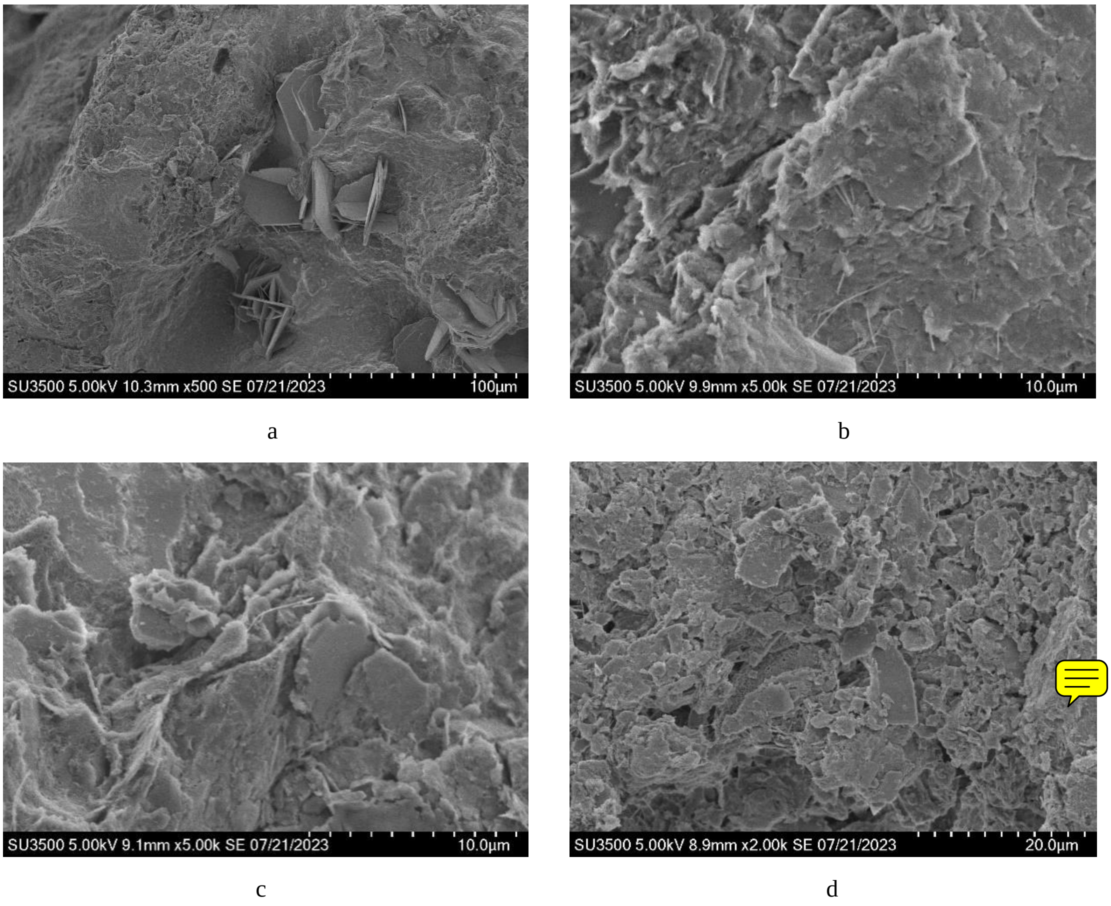

a—0%, b—10%, c—30% , d—50%

Fig 9. SEM photographs of the specimens with different proportions of slag as a curing agent

3 Conclusions

(1) The density of lightweight soil is mainly affected by the EPS content and decreases nonlinearly with the increase of EPS content, and the decrease is gradually decreasing.

(2) The UCS decreases nonlinearly with increasing EPS content; increases linearly with increasing curing agent content; and increases with increasing slag content, with the highest strength at 30% slag content, and the strength of the lightweight soils decreases beyond 30%.

(3) Lightweight soils with different ratios show two types of hardening and softening under

different confining pressures. The triaxial stress-strain characteristics are essentially determined by the combined effect of the material ratio, which determines the strength of the cemented structure, and the confining pressure, which determines the stress state.

(4) The lamellar and reticular cementation structure formed by the internal hydration reaction of lightweight soil is the main source of its strength, which directly affects the mechanical properties of lightweight soil. Compared with the lightweight soil without slag, the lightweight soil with the addition of an appropriate amount of slag, the integrity of the soil body is enhanced, and the strength is increased.

References

1. Sreerekha S, Kumar PR. Properties and Application of EPS beads as aLightweight Fill Material for EmbankmentConstruction. International Journal of Innovative Research in Science, Engineering and Technology. 2014;3.
2. Gao H, Liu J, Liu H-l. Geotechnical properties of EPS composite soil. International Journal of Geotechnical Engineering. 2011;5:69 - 77. https://doi.org/10.3328/IJGE.2011.05.01.69-77
3. Miao L, Wang F, Lu W, Li J. Study on Cement-Treated Yangtze Hydraulic Sand Mixed with Expa nded Polystyrenes (EPS) Beads as Backfill Material in Highway Embankments. 2010. https://doi.org/10.1061/41104(377)46
4. Khajeh A, Jamshidi Chenari R, Payan M. A Review of the Studies on Soil-EPS Composites: Beads and Blocks. Geotechnical and Geological Engineering. 2020;38:3363-83. https://doi.org/10.1007/s10706-020-01252-2
5. Beju YZ, Mandal JN. Expanded Polystyrene (EPS) Geofoam: Preliminary Characteristic Evaluation. Procedia Engineering. 2017;189:239-46. https://doi.org/10.1016/j.proeng.2017.05.038
6. Li M, Wen K, Li L, Tian A. Mechanical properties of expanded polystyrene beads stabilized lightweight soil. Geomechanics and Engineering. 2017;13:459. https://doi.org/10.12989/gae.2017.13.3.459
7. Feng Y. Experimental research on the compressive strength influencing factors of expanded polystyre ne sheet silt mixed lightweight soil material. Materials Research Innovations. 2015;19:S10-64 - S10-68. https://doi.org/10.1179/1432891715Z.0000000002089
8. Lu W-H, Wang J, Zhang Y. Strength Characteristics of Cement Treated and Expanded Polystyrene Mixed Lightweight of Waste Soil from the Construction Site of a Yangtze River Bridge in China. Advances in Civil Engineering. 2020;2020:1-7. https://doi.org/10.1155/2020/3640510
9. Feng Y, Pei YB. Study on the Stress and Strain of EPS Silt Light-Weight Soil by Laboratory Test. Applied Mechanics and Materials. 2014;580-583:499 - 502. https://doi.org/10.4028/www.scientific.net/AMM.580-583.499
10. YI Yao-lin, QING Xue-wen, ZHUANG Yan, LIU Song-yu, DU Guang-yin. Utilization of GGBS in stabilization of soft soils and its mechanism. Chinese Journal of Geotechnical Engineering, 2013, 35(S2): 829-833. China.
11. Gopal CV, Suresh, Nath VG. Partial Replacement of Cement with GGBS in Concrete. International Journal of Advance Research, Ideas and Innovations in Technology. 2017;3:313-22.
12. Barsagade DM, Patil KS, editors. Stabilization of Soft Soil With Granulated Blast Furnace Slag and Fly Ash. 2018. https://doi.org/10.15623/ijret.2013.0202005
13. Loke CK, Lehane B, Aslani F, Majhi S, Mukherjee A. Non-Destructive Evaluation of Mortar with Ground Granulated Blast Furnace Slag Blended Cement Using Ultrasonic Pulse Velocity. Materials. 2022;15. https://doi.org/10.3390/ma15196957
14. Saha S. A Study on Partial Replacement of Cement with Rice Husk Ash & Fine Aggregates with Ground

Granulated Blast Furnace Slag. 2022.

15. Usmi S, Kumar RV. Experimental Investigation of Concrete using Partial Replacement of Cement with GGBS and Coarse Aggregate with Demolishing Waste. International journal of engineering research and technology. 2018;3.

16. Zhao Qingxin, Cai Hongwei, An Sai, et al. Strength and Mechanism of Stabilized Coastal Saline Soil by Cement and Fine Slag. Journal of Building Materials, 2020, 23(03): 625-630. China. http://dx.doi.org/10.3969/j.issn.1007%209629.2020.03.020

17. Liang SH, Zhou SZ. Experimental research about mechanical properties and microscopic structures of Nansha soft soil stabilised with slag and cement. Materials Research Innovations. 2015;19:S10-69 - S10-74. https://doi.org/10.1179/1432891715Z.0000000002090

18. Nu NT, Son BT, Hai PV, Geology V. Utilisation of ground granulated blast furnace slag (GGBFS) for soft soil improvement by deep mixing method. 2020. https://doi.org/10.46326/JMES.2020.61(1).10

19. Lal BRR, Badwaik VN. Experimental Studies on Bottom Ash and Expanded Polystyrene Beads–Based Geomaterial. Journal of Hazardous, Toxic, and Radioactive Waste. 2016;20:04015020. https://doi.org/10.1061/(ASCE)HZ.2153-5515.0000305

20. Deshmukh R, Iyer S, Bhangare P. Geotechnical characterization of Expanded polystyrene (EPS) beads with industrial waste and its utilization in flexible pavement. Materials Today: Proceedings. 2021. https://doi.org/10.1016/j.matpr.2021.07.462

21. GB/T 50123-2019, Standard for geotechnical testing method. China.

22. MEI Lifang, XU Guangli. Preparation and mechanical properties of fiber expanded polystyrene particle lightweight soil. Acta Materiae Compositae Sinica, 2016, 33(10): 2355-2362. China. https://doi.org/10.13801/j.cnki.fhclxb.20160621.001

23. Hou T-s. Prescription Formula of Foamed Particles in Lightweight Soil. Geotechnical and Geological Engineering. 2015;33:153-60. https://doi.org/10.1007/s10706-014-9814-z

24. LIU Hanlong, DONG Jinmei, ZHOU Yundong, et al. Study on the stress-strain characteristics of light heterogeneous soil mixed with expanded polystyrene. Chinese Journal of Geotechnical Engineering, 2004, 26(5): 579-583. China.

25. PEI Zhenwei, HOU Tianshun, LUO Yasheng. Experimental study on unconfined compressive strength of soil and EPS particles mixtures. Journal of Engineering Geology, 2018, 26(6): 1454-1462. China. https://doi.org/10.13544/j.cnki.jeg.2017-452

26. HOU Tianshun. Experimental Study on Mechanical Properties of Foamed Particle Light Weight Soil Mixed with Silt. China University Of Geosciences, 2008. China.

27. DONG Jin-mei, LIU Han-long, HONG Zhen-shun, et al. Test study on compression and deformation behavior of light heterogeneous soil mixed with expanded polystyrene. Rock and Soil Mechanies, 2006, 27(2): 286-289. China.

28. Cui C, Yu C, Zhao J, Zheng J. Steel Slag/Precarbonated Steel Slag as a Partial Substitute for Portland Cement: Effect on the Mechanical Properties and Microstructure of Stabilized Soils. KSCE Journal of Civil Engineering. 2022;26:3803 - 14. https://doi.org/10.1007/s12205-022-1762-1

29. Li Q, Li B, Li X-m, He Z, Zhang P. Microstructure of pretreated steel slag and its influence on mechanical properties of cement stabilized mixture. Construction and Building Materials. 2022. https://doi.org/10.1016/j.conbuildmat.2021.125799

script

Click here to access/download
Supporting Information - Compressed/ZIP File Archive
data.rar
