# Supplementary material for: Research on the mechanical properties of EPS lightweight soil mixed with slag
Source: PLoS One. 2024 Jan 24;19(1):e0297372. doi: 10.1371/journal.pone.0297372 (PMC10842304; doi:10.1371/journal.pone.0297372)
Supplement: Attachment — Submitted filename: Response to Reviewers.docx [file pone.0297372.s004.docx]

Dear Editors and Reviewers:

Thank you for your letter and for the reviewers’ comments concerning our manuscript entitled "Research on Mechanical Properties of Slag EPS Mixed Lightweight Soil"(ID: PONE-D-23-31063). Those comments are all valuable and very helpful for revising and improving our manuscript, as well as the important guiding significance to our research. We have studied comments and have made revision carefully. Revised portions are marked in red. We sincerely hope this manuscript will be finally acceptable to be published. Thank you very much for all your help.

Please find the following Response to the comments of reviewers:

Reviewer #1:

* English needs to be improved

Response: Thanks for your kind reminder. We have carefully checked the spelling, language, grammar, and punctuation, corrected some errors, and polished the whole manuscript. We hope our manuscript can meet the journal’s standard.

* EPS definition need to be given in abstract

Response: Thank you for your careful review and valuable comments. we have added the EPS definition. “Expanded polystyrene (EPS)” has been given in abstract.

* particle size and their % need to be given in abstract

Response: Thanks so much for your suggestion. we have added the information about particle size and their % in abstract. The particle sizes of EPS beads are 1~3mm，the EPS content are 1%, 2%, 3%, 4%, the slag-cement composite binding agent are 10%, 15%, 20% and 25%, respectively. on line 14-16.

*Rewrite paragraph on line 36

Response: Thanks so much for your comment. We have re-written paragraph on line 36, and this section is much better.

* Rewrite section 2.1 to be more understandable

Response: Thank you for your careful review and valuable comments. We have re-written section 2.1, and this section is more understandable.

* line 141. unlimited increase? what do you mean?

Response: Thanks so much for your comment. With the increase of EPS beads, the density of lightweight soil will decrease, too much EPS is difficult to make a specimen, the strength of lightweight soil will also decrease, The EPS content cannot be unlimited increased, a suitable ratio is needed to meet the needs of the project.

* Table 3 scheme need to be capital letter and why 20% curing agent is decided to be worked on?

Response: Thank you for your careful review and valuable comments. We have modified the letter in Table 3, the word "curing agent" has been replaced with "binding agent". the 20 % binding agent is a typical representative.

* it may be better to have 3D- with 2 y axes to see effect of agent and EPS graphs on results

Response: Thanks so much for your suggestion. based on your suggestion, we have added 3D- with 2 y axes to see effect of agent and EPS graphs, this section is more specific and clearer. on line 236

* line 258. cut line? rewrite the sentence

Response: Thanks so much for your comment. We are very sorry for our incorrect writing, the "cut line" means Secant, we have corrected the words.

* line 303 physic mechanical?

Response: Thank you for your careful review and valuable comments. English expression error, replace " physic mechanical " with " physical and mechanical properties ".

* line 310 why those % values are chosen for SEM

Response: Thanks so much for your comment. In microscopic test, the main observation is the state of EPS, its % value does not affect its state, a representative % value can be chosen, 20% binding agent is a suitable % value for microscopic test.

* in conclusion part, after giving results, conclusion need to be given to understand why this study has been conducted

Response: Thank you for your careful review and valuable comments. The significance and value of conducting this study is given after the conclusion. on line 388-390.

Thank you again for your positive and constructive comments and suggestions on our manuscript. We tried our best to improve the manuscript and made some revisions in the manuscript. We hope you can accept our revised manuscript.

Reviewer #2:

1-Language and Terminology needs rigorous improvement. Some of the sentences appear to be unclear or ambiguous. The language used must be precise and that technical terms must be appropriately defined and explained. A formal proof read is necessary for the whole manuscript. Some of the ambiguous parts are highlighted but the rest also need checking. A format check is also strongly advised.

Response: Thanks for your kind reminder. We have carefully checked the spelling, language, grammar, and punctuation, corrected some errors, and polished the whole manuscript. Technical terms are given appropriate definitions and explanations. We hope our manuscript can meet the journal’s standard.

2-The novelty and the research question of the work is unclear. Why and where do we need light soil and in which structures it could be applied? What is the application? What is the contribution of the research and its novelty in the field of geotechnical engineering. What specific gap does this study fill, and how does it advance the current understanding of the subject?

Response: Thank you for your careful review and valuable comments. on line 84-93, I have modified and supplemented the above formulation. Lightweight soil is widely used as a new lightweight fill material due to its light weight and high strength. Previous studies focus on the behavior of various soils mixed with EPS beads and cement. Slag is a common building material used in engineering and construction, mixing slag in EPS lightweight soil can reduce the amount of cement, lower filling costs, and improve the strength of lightweight soil, achieving economic and environmental protection. Currently there is a lack of research on EPS lightweight soil mixed with slag, the slag will be developed into a high value-added filling materials to achieve large-scale utilization of slag, accelerate the transformation and upgrading of waste utilization, and produce a new type of lightweight filling material. Therefore, it is necessary to carry out research on the mechanical properties of EPS lightweight soil mixed with slag.

3- The methodology needs improvement. Which standards are used? How the EPS is prepared? What are the properties of EPS? What type of clay is used?

Response: Thanks so much for your comment. on line 105, 107-108, 135-136, 147-149, I have modified and supplemented the above formulation and other relevant places. The standard is " Standard for Geotechnical Test Methods (GB/T-50123-2019)". The lightweight materials are EPS spherical beads, produced by Plastic Foam Factory, diameter 1~3mm, pure particle density of 0.024g/cm^3^, and packing density of 0.016g/cm^3^, with light weight, seismic, low cost, etc. EPS added to the soil body can significantly reduce the soil body's own load, to achieve the role of reducing soil pressure and load. The original soil used in the test is silty clay.

4- The clay here seems to CL (?), it is not clear why it needed the improvement at the first place.

Response: Thanks so much for your comment. The original soil in this case is silty clay. The subject of our research is lightweight soil, a new type of artificial fill material. on line 37-38, 40-41, 45-51, I have modified and supplemented the above formulation and other relevant places. With the increase of EPS beads, the density of lightweight soil is greatly reduced, generally up to 1.2g/cm3 or less. The mixing of binding agent makes the lightweight soil have certain strength and deformation resistance. In order to improve the applicability of lightweight soil, to facilitate the use of local materials, to reduce transportation and construction costs, the waste soil excavated from the pit of the nearby construction site was selected as the original soil, mixed with EPS beads and binding agent, and made into EPS lightweight soil. Due to its light weight and high strength, it plays a vital role in weak foundations, preventing highway settlement, stabilizing slopes, backfilling bridge abutments and underground cavities.

5- The addition of EPS seems to decrease the strength of samples, how the usage of EPS is justified then?

Response: Thank you for your careful review and valuable comments. EPS has the advantages of light weight, seismic, low cost, etc., Adding EPS can reduce soil density, soil pressure, and settlement. Although the strength is reduced, adding binders can compensate for the loss of strength, the strength of lightweight soil can be adjusted according to engineering needs. The use of EPS is reasonable.

6- There isn't a comparison with the original soil with no additive, it's likely that the soil with no admixture would exhibit better properties. A comparison with the plain soil must be added.

Response: Thanks so much for your suggestion. based on your suggestion, we have added Fig. 2 about the original soil with no additive on line 181.

7- what is it meant by curing agent? Slag and cement are most commonly used as binding agents.

Response: Thanks so much for your comment. English expression error, replace " curing agent " with " binding agents ".

8-EPS could may cause deformation and settlement within time and loading, how the authors justify the usage of it?

Response: Thank you for your careful review and valuable comments. Researches have conducted relevant research on EPS lightweight soil, shown that the addition of EPS to soil can reduce soil's density and weight, but the addition of binders, EPS lightweight soil has a certain strength and deformation resistance. Due to EPS lightweight characteristics, it reduces soil load and pressure, instead reduces deformation and settlement.

9- Is there any environmental effects of using EPS? slag within the soil? It most be considered.

Response: Thank you for your careful review and valuable comments. In 34-35, 68-72, I have modified and supplemented the above formulation and other relevant places. EPS molecular structure is stable, does not release harmful substances and cause chemical reactions to the soil. Researches analyzed the composition and properties of slag, and studied the application of slag in the fields of building materials, sewage treatment, agriculture and resource utilization. The results show that making full use of the secondary resources of slag can improve the protection of the environment, realize the harmless treatment of slag, and further improve the residual value of slag.

Thank you again for your positive and constructive comments and suggestions on our manuscript. We tried our best to improve the manuscript and made some revisions in the manuscript. We hope you can accept our revised manuscript.
